# Performance Evaluation and Action Mechanism Analysis of a Controllable Release Nanocapsule Profile Control and Displacement Agent

**DOI:** 10.3390/polym15030609

**Published:** 2023-01-24

**Authors:** Fang Shi, Jingchun Wu, Zhongcheng Li, Bo Zhao, Jian Li, Shenglan Tang, Weizhi Tuo

**Affiliations:** 1 Key Laboratory for EOR Technology (Ministry of Education), Northeast Petroleum University, Daqing 163318, China; 2 PetroChina Jilin Oilfield Co., Exploration and Development Research Institute of Jilin Oilfield Branch Songyuan, Songyuan 138000, China; 3 Daqing Oil Field Co., Ltd., No. 6 Oil Production Plant, Daqing 163000, China; 4 PetroChina Tarim Oilfield Company, Korla 841000, China

**Keywords:** intelligent profile control agent, controlled release, nanocapsule, indoor performance evaluation

## Abstract

With the acceleration in oilfield developments, reservoir advantage channels have been gradually developed. This has led to ineffective circulation in the oilfield injection system and a significant decrease in production. The profile control and displacement technology of low-permeability and heterogeneous reservoirs are in urgent need of updating. In this paper, an intelligent profile control and displacement agent is proposed. The controlled release mechanism and profile control and displacement mechanism is clarified by physical simulation experiments. The profile control agent is a nanocapsule with environmental response and controlled release. The structure of the capsule is a core–shell structure, which is composed of an amphiphilic copolymer AP-g-PNIPAAM and Janus functional particles. The surface chemical stability of the micro/nanocapsule is analyzed by a potentiometric method. The study shows that a temperature at 45 °C causes a potential change in the micro/nanocapsule, indicating that the micro/nanocapsule has a slow release at this temperature. When the temperature is in the range of 40 to 45 °C, the absorbance greatly increases; therefore, it is considered that the capsule wall LCST is about 45 °C. Heating causes the surface contraction of the capsule wall to intensify, the micropores in the capsule wall to increase, the release amount to increase and the release rate per unit time to increase. The release time increases proportionally with the increase in capsule wall thickness. When the release time is the same, an alkaline or acidic environment can improve the release rate of the nanocapsule. The effect of profile control and flooding is evaluated through different differential core models. The research shows that the controlled release micro/nanocapsule has a good environmental response and the internal components can be effectively controlled by adjusting the temperature or pH value. This research has shown that the nanocapsules have good application prospects in low-permeability heterogeneous reservoirs.

## 1. Introduction

During the development of low permeability reservoirs, which is affected by dominant seepage channels, heterogeneity and fractures, water levels rise rapidly, leading to the restricted development of the oil production rate and a low recovery degree [1]. There is still a large amount of oil remaining in the late stage of water flooding. Based on improving the plane and microheterogeneity of water flooding and realizing the technical requirements of fluid flow diversion in oil layers, it is necessary to carry out effective profile control and displacement treatment of reservoirs [2,3,4,5]. At present, polymer microspheres and polymer capsules are mainly used for plugging to induce a diversion of the fluid flow of the displaced medium, and then chemical displacement agents are added for oil displacement [6,7,8,9,10,11,12,13,14,15,16,17]. The application of polymer capsules can effectively block high permeability layers—even high permeability bands or large pores—and increase the utilization of medium and low permeability layers. However, field tests have also shown that during the injection of polymer capsules into underground reservoirs, factors such as the high viscosity and large molecular size of the polymer capsule will cause wellbore plugging and the pollution of the low and medium permeability layers. In actual field tests, the effect of polymer capsules is also affected by the reservoir’s physical properties, the concentration of polymer and crosslinking agent, the types of chemical agent and other factors. According to actual needs, some polymer capsules also need to be unblocked [10,11]. The current method of comprehensive profile control and use of displacement agents, whether focusing on profile control or oil displacement technology, has certain limitations. Only a combination of the two can achieve the dual effects of profile control and oil displacement.

In order to improve the plugging effect in the dominant leaking area, controllable release micro/nanocapsules and displacement agents with low initial viscosity and environmental response have been screened and evaluated in the laboratory. The results show that intelligent nanocapsule systems can effectively block the water channel, can change the flow direction of the injected fluid, can improve the reservoir heterogeneity, can improve the sweep and oil displacement efficiency and have the potential to improve the recovery of low-permeability reservoirs [12].

**Figure 1:**
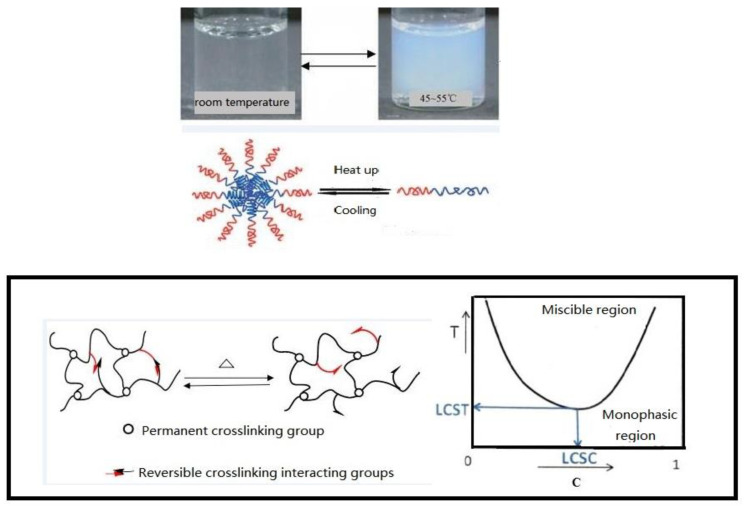
Comparison of the temperature-sensitive response.

Environmental response-controlled nanocapsules are a matrix with fully dispersed and uniform effective components. The principle of controlled release mainly includes five forms: diffusion (1), dissolution (2), dissolution (3), osmotic pressure (4) and ion exchange (5) [13,18,19,20]. Of these, (1), the diffusion principle, is the process in which the effective components are first dissolved and then diffused out of the nanocapsule. (2) is the dissolution principle, which requires that the solubility of the effective components is significantly reduced in advance so that a concentration difference is formed in the release process, so as to increase the particle size of the nanocapsule, reduce the dissolution rate and realize controlled release. (3), the principle of dissolution, is the dual function of diffusion and dissolution. (4), the principle of osmotic pressure, is that a polymer is used as the coating on the outside and the core material is water-soluble. The coating forms a semi permeable membrane, and water can penetrate the membrane to maintain the saturation state of the internal components, so as to achieve the effect of controlled release. (5), the principle of ion exchange, requires the exchange of components in the inner and outer space and the release of effective components after this exchange. Generally speaking, the release can be summarized as the way in which the permeability is changed or the permeable membrane is dissolved or degraded by water immersion or heating [21,22]. Considering that the pore throat size matching problem exists in the migration of nanocapsules in porous media, the sensitive response of nanocapsules is not only the temperature response of the target layer of the reservoir, but also the timed dissolution response formed by coordinating and regulating the thickness of the capsule wall. After a nanocapsule is injected from the wellhead, and before it has responded to the temperature, it stays at the position with relatively small pore throat size [23,24]. After a certain amount of time, the capsule wall dissolves, so that a blocking phenomenon is observed. Temporary delays to this process can also play a role in expanding the swept volume and play a positive role in improving the subsequent displacement efficiency. Using supramolecular interactions or dynamic covalent bonds as molecular switches can temporarily alter the shape of a nanocapsule. As shown in Figure 1, when the ambient temperature reaches the lower critical solution temperature (LOST), the chemical stability of the outer surface of the core–shell structure changes. When the temperature is higher than the LOST, hydrophobic association occurs in the side chain of the polymer, which is hydrophobic. At this time, the polymer as a whole is amphiphilic [25]. At this time, due to physical crosslinking, the polymer has shear thickening and good salt resistance.

The release mechanism is shown in Figure 1. Based on the original intention of preparing the target nanocapsule, the core–shell structure and shell are dissolved, the effective components are dissolved into solution, and then are diffused out of the nanocapsule into the porous medium. The release rate of the nanocapsule is affected by several main factors; (1) the core material solubility; (2) the core material diffusion coefficient; (3) the shell properties and thickness; and (4) the release environmental temperature. The nanocapsule shell developed in this paper is an amphiphilic copolymer, AP-g-PNIPAAM, which has a certain viscosity and can emulsify with crude oil. Janus functional particles are embedded in the amphiphilic AP-g-PNIPAAM, and AP-g-PNIPAAM gradually dissolves at the response temperature, releasing at a constant rate with time. Based on the above analysis, it is necessary to clarify the release characteristics of the nanocapsule in porous media.

## 2. Material and Methods

### 2.1. Experimental Materials

A crude oil from one of the oil production plants in Daqing, China (45 °C simulated oil viscosity 9.8 MPa·s) was used for this experiment. The salinity of simulated water used in the experiment was 4500 mg/L. The nanocapsule dispersion (PG) was provided by the Key Laboratory of EOR of the Ministry of Education of Northeast Petroleum University (developed by the first author of this manuscript). The preparation method was as follows: firstly, the polymer main chain P (AM-DMAAC-AA) was prepared by fine lotion polymerization. Azobutyronitrile (0.1 wt% AIBN) was used as an initiator in the experiment and n-dodecyl mercaptan (0.2 wt% DDM) was used as a relative molecular weight regulator (0.1 wt%—n SDS:n OP-10 = 1:2). Hexadecyl dimethyl allyl ammonium chloride was used as the ligand, acrylamide monomer (AM) was used in a given proportion, as was sodium acrylate (NaAA). The chemical reagents are all from the brand of Chinese medicine Xilong Kemio, Tianjin, China. Cetyldimethyl allyl ammonium chloride (C16-DMAAC) (n AM:n NaAA:n C16-DMAAC = 1:0.3:0.8) was reacted by atom transfer radical polymerization (ATRP) at 60 °C and at pH 6–8 to obtain P (AM-DMAAC-AA). Then, it was grafted with N-isopropylacrylamide prepolymer with carboxyl terminating groups (PNIPAAM) to obtain an amphiphilic, temperature-sensitive graft copolymer with controllable molecular weight. The polymer was amphiphilic and could be assembled into molecules in water to form nanocapsules. The size of the nanocapsules prepared in this paper ranged from 100 nm to 300 nm; the critical dissolution temperature was about 45 °C. Afterwards, the nanocapsules continued to react with Janus functional particles in a certain proportion to generate Janus smart nanocapsules. The synthesis of Janus functional particles was as follows: 0.5 g SiO_2_ nanoparticles and 3 g solid paraffin were dispersed in 20 g deionized water and emulsified for 1 h in a high-speed mixer at 2800 r/min. After emulsification, the emulsion was placed in a refrigerator for cooling to solidify the paraffin. To remove the un-adsorbed nano SiO_2_ particles, the surface of the solidified paraffin emulsion was washed with drops with deionized water and then the solidified paraffin emulsion drops were dried under vacuum at 45 °C. In a typical silylation process, the dried and solidified paraffin emulsion drops were dispersed to a concentration of 0.1% γ-MPS in ethanol at room temperature for 72 h. Then, to filter out the paraffin emulsion drops, the surface of the paraffin emulsion was washed with drops with ethanol to remove the unreacted γ-MPS and unadsorbed SiO_2_ particles. Then, the paraffin was dissolved in chloroform, the released modified SiO_2_ particles were collected through centrifugal rinsing, and they were dried in a vacuum drying oven for 24 h. In this paper, the oil-water interfacial tension was 8 × 10^−3^ mN/m when the Janus functional particle concentration was 0.2 wt%, the temperature was 45 °C, salinity was 4500 mg/L and the crude oil viscosity was 9.8 mPa·s. This work was reported by the first author in the *Journal of Materials* [8].

The particle size range of PG was 100 nm to 300 nm. It had both lipophilic and hydrophilic characteristics and temperature- and pH-responsive characteristics.

### 2.2. Structural Characterization by Infrared Spectroscopy

**Figure 2:**
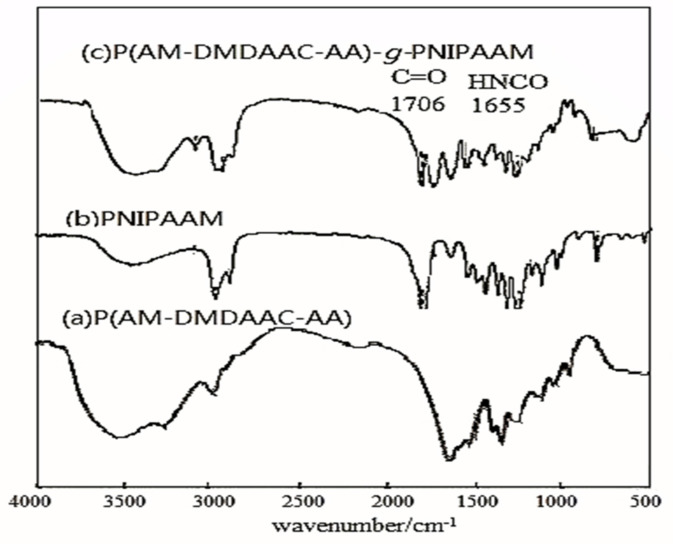
Infrared spectrum.

As shown in Figure 2, the characteristic absorption peak of –NH_2_ can be found in the infrared spectrum of the sample at 3426 cm^−1^ and 3193 cm^−1^. The characteristic absorption peaks of the N-H bending vibration are 1617 cm^−1^ and 1561 cm^−1^. The bending vibration absorption peak of the methyl group is 1453 cm^−1^. The characteristic absorption peaks of the methylene stretching vibration are 2932 cm^−1^ and 2853 cm^−1^. The characteristic absorption peak of the C = O in amide and –COO–is 1667 cm^−1^. The C-N stretching vibration absorption peak in amide group is 1401 cm^−1^. The C-O stretching vibration absorption peak in carboxylic acid is 1321 cm^−1^. The stretching vibration absorption peak of the long chain alkyl group is 110 cm^−1^. Therefore, the polymer can be qualitatively considered as the target copolymer P(AM-DMAAC-AA). The target product, AP-g-PNIPAAM, was obtained by combining the spectral positions of the characteristic peak C = O of N-isopropylacrylamide and HNCO.

### 2.3. Apparatus and Experimental Procedures

There is an electrostatic interaction between PG particles, and the electrostatic repulsion is conducive to the uniform dispersion of the micro/nanocapsules in the dispersion medium. When the surface potential of PG changes, this can affect embedding and release. Zeta potentiometry was used to characterize the release times of PG. When the potential mutation point of the dispersion system appeared, it was considered that the effective component of capsule was released.

**Figure 3:**
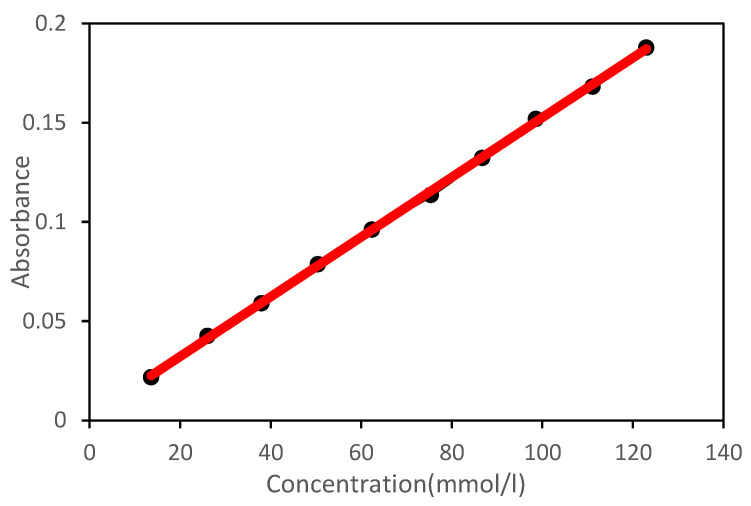
Janus concentration release standard curve.

In the target reservoir, when the response temperature was reached, although the shell switch was turned on, it only triggered the swelling or hydration of the shell. The core material was released from it regularly with the expansion of the shell. The controlled release mechanism can be characterized in two ways: first, the swelling rate can be expressed in Formula (1). Second, the concentration can be measured by spectrophotometry, and the relationship between the concentration and various influencing factors can be calculated. The standard curve obtained is shown in Figure 3.

(1) $$\eta =\frac{d_{2}-d_{1}}{d_{1}}$$

*η* indicates swelling rate,

*d*_2_ represents the median particle size after swelling in nm;

*d*_1_ represents the median particle size before swelling in nm.

## 3. Results and Discussion

### 3.1. Characterization of Surface Potential of Nanoparticles

**Figure 4:**
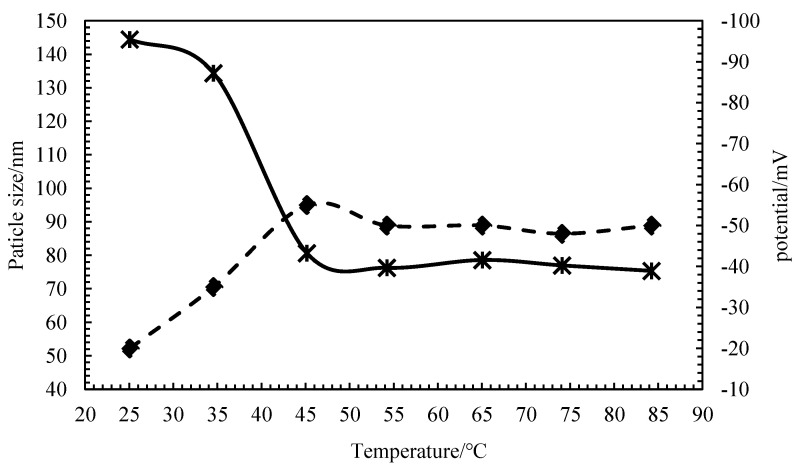
Influence curve of temperature on dispersion stability. (**––––** particle size; **– – –** potential).

The prepared nanocapsule was made from an amphiphilic, temperature-sensitive graft copolymer P(AM-DMAAC-AA)-g-PNIPAAM (PG for short). As shown in Figure 4, the absolute value of nanocapsule potential increased and the particle size decreased with increasing temperature. Until a temperature of about 45 °C, the change tends to be stable. It shows that the nanocapsule is a slow-release nanocapsule under this temperature. Janus functional particles enter the molecular gap in the solution, and the PG shell is temperature responsive. The temperature has little effect on the particle size and potential change in Janus functional particle dispersion, indicating that Janus functional particles have a good temperature resistance. The stability of the particles is also good at temperatures between 45 °C and 85 °C.

**Figure 5:**
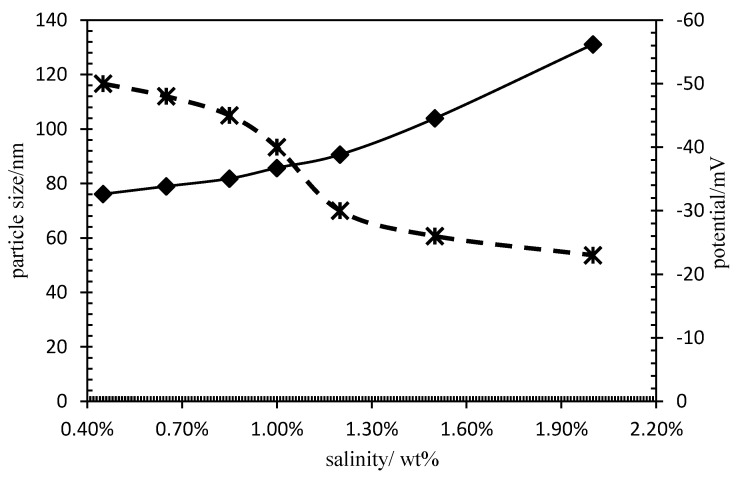
Influence curve of salinity on dispersion stability. (**––––** particle size; **– – –** potential).

At about 45 °C, PG releases Janus functional particles, and the solution system is mainly composed of a Janus functional particle dispersion. An analysis of the influence of mineralization on particle stability is shown in Figure 5. With the increase in mineralization, the particle size gradually increases and the absolute value of potential decreases. The reason for this phenomenon is that the high concentration electrolyte is distributed around the particle double electric layer. Based on the electrostatic effect, the double-electric layer is compressed, which reduces the electrostatic repulsion between particles and affects the particle dispersion and agglomeration. Through data analysis, under the condition of high salinity, the particle size is still in the nanometer range. Therefore, Janus functional particles potential salt-resistant properties.

### 3.2. Characterization of Capsule-Controlled Release Mechanism

#### 3.2.1. Effect of Salinity on the Swelling Rate

**Figure 6:**
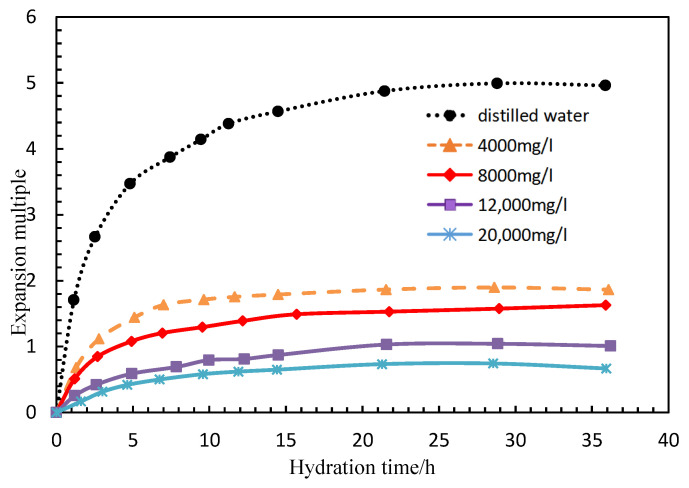
Effect of salinity on swelling rate.

The experimental parameters are shown in Table 1. PG (0.15 wt%), with a thickness of 14 nm, was placed in aqueous solutions with different salinities at 45 °C (pH = 7) and the particle size of PG at different times was measured. The swelling rate was calculated. As shown in Figure 6, the higher the salinity, the slower the swelling rupture time. The swelling rate (with a salinity of no less than 0.15 wt%) was basically saturated between 10 h and 15 h, and it swelled to between 0.5 times and 2 times the normal size. When the salinity was 4000 mg/L, it took 15 h for the swelling to reach saturation, and the expansion reaches 1.5 times of the normal size.

#### 3.2.2. Effect of Temperature on the Swelling Rate

**Figure 7:**
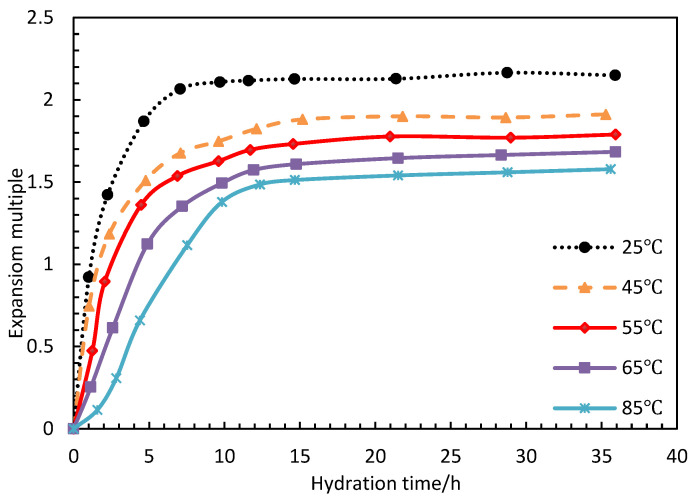
Effect of temperature on swelling rate.

The experimental parameters are shown in Table 2. PG (0.15 wt%), with a shell thickness of 14 nm, was placed in a dispersion system with a salinity of 0.5 wt% and a pH value of 7 in a water bath at different temperatures, and the particle size of PG at different times was measured. The swelling rate was calculated. As shown in Figure 7, the higher the temperature, the slower the swelling rupture time. The swelling rate of PG above 45 °C basically reached saturation within 5 h, and the swelling rate was between 1.5 and 2. The swelling rate was started within 15 h at 45 °C.

#### 3.2.3. Effect of pH Value on the Swelling Rate

**Figure 8:**
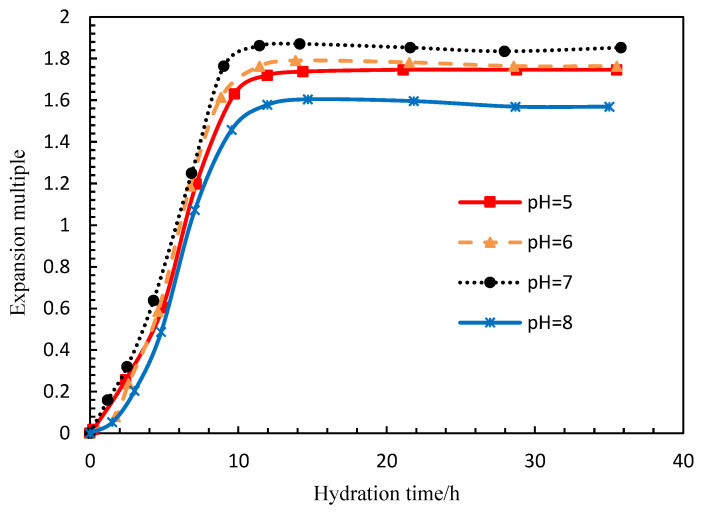
The effect of pH value on the swelling rate of the capsule.

The experimental parameters are shown in Table 3. With the increase in time, the shell thickness changed slowly. When it swelled to about 1.5 times the particle size of PG (150 nm to 450 nm), this is the starting point of release. The injection rate was constant. Taking a PG dispersion system with a thickness of 14 nm, a concentration of 0.15 wt%, a salinity of 0.5 wt%, and pH value of 7 as an example, the interval between PG injection and the starting point of release was 5 h. By adjusting the thickness, the starting time of release can be adjusted. As shown in Figure 8, the pH value has little effect on the expansion rate. Increasing or decreasing the pH value will slightly reduce the expansion. Studies have shown that the starting time of PG controlled release can also be shortened or prolonged by adjusting the temperature, salinity and pH value.

#### 3.2.4. Influence of Shell Thickness on the Release Rate

**Figure 9:**
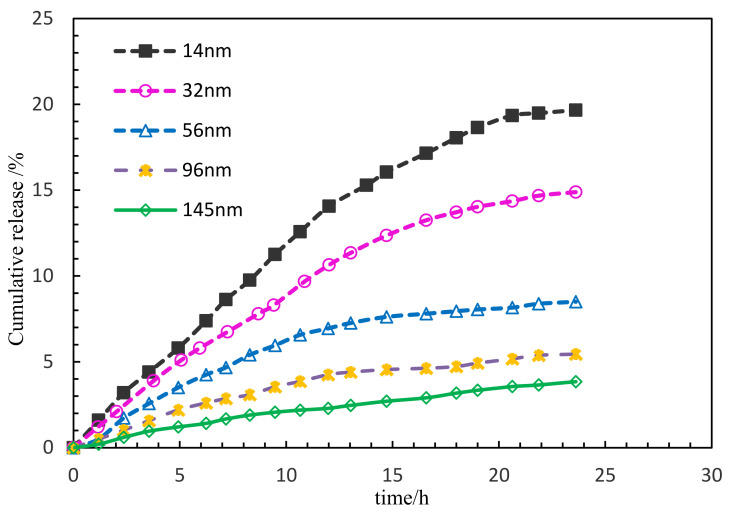
The changes in release rate of the PG sample with time.

The experimental parameters are shown in Table 4. The release rate of PG samples prepared with different shell thickness was tested with time. Different thicknesses of 14 nm, 32 nm, 56 nm, 96 nm and 145 nm were tested with a PG with concentration of 0.15 wt%, a pH of release medium of 7 and a temperature of 45 °C. As shown in Figure 9, the slope of the release rate versus time curve changed slightly, and is almost a constant rate process. The results showed that the cumulative release rates of 24 hpg were 19.66% at 14 nm, 14.89% at 32 nm, 8.50% at 56 nm, 5.45% at 32 nm and 3.85% at 145 nm, i.e., with the same release time, the cumulative release rate decreases with the increase in thickness.

#### 3.2.5. Effect of pH Value on the Release Rate

**Figure 10:**
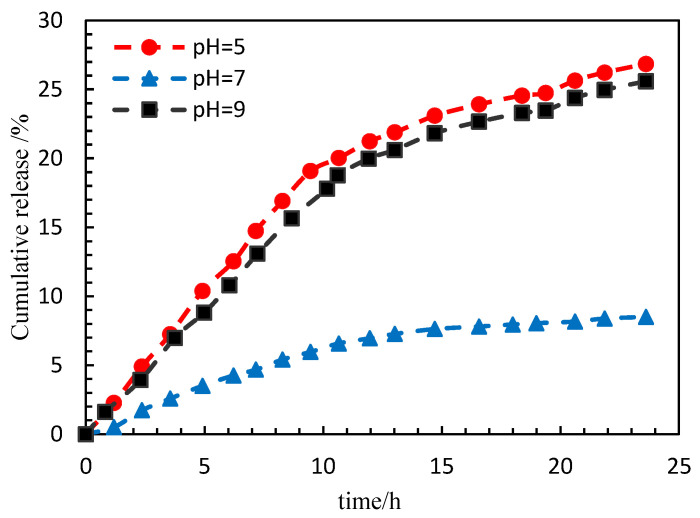
Release rate of PG samples changes with time.

The experimental parameters are shown in Table 5. The release rate of PG samples prepared under different pH conditions was tested with time. The test temperature was 45 °C, the pH values were 5, 7 and 9 and the PG shell thickness was 56 nm. As shown in Figure 10, the slope of the release rate versus time curve changed slightly, and is almost a constant rate process. When the pH value was 7, the cumulative release rate of PG in 24 h was about 8.5%; at the same release time, it can be found that under acidic or alkaline conditions, the cumulative release rate of PG was increased, the cumulative release rate in 24 h was close to 25% and the increase range was simple. Therefore, the release can be shortened by reducing or increasing the pH value of the PG dispersion.

#### 3.2.6. Effect of Temperature on the Release Rate

**Figure 11:**
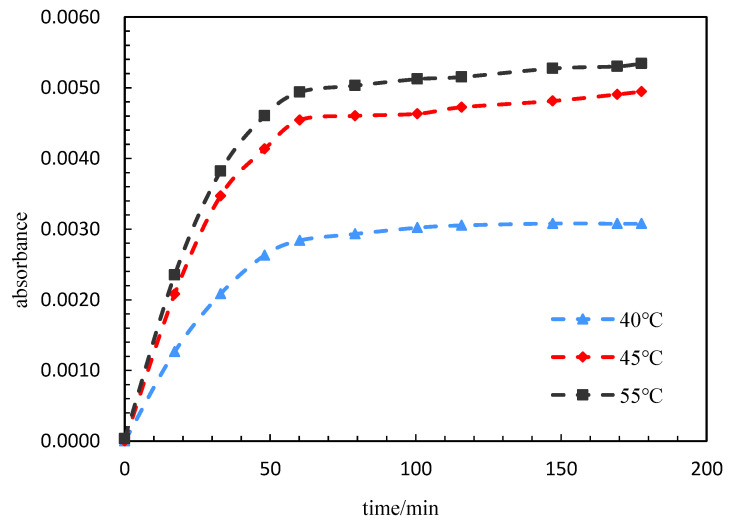
Absorbance value of PG sample with time.

The experimental parameters are shown in Table 6. The absorbance of PG samples was tested with time at different temperatures. Experimental temperatures of 40 °C, 45 °C and 55 °C were testes with a PG thickness of 14 nm. As shown in Figure 11, the absorbance increased with the increase in temperature after the same amount of time. When the temperature was in the range of 40 to 45 °C, the absorbance increased greatly; therefore, it is considered that the LCST of the shell was about 45 °C. As the temperature continued to rise, the absorbance increased. It shows that the temperature rise intensified the decrease in the shell surface, enlarged the micropores in the capsule wall, increased the release amount and increased the release rate per unit time.

**Figure 12:**
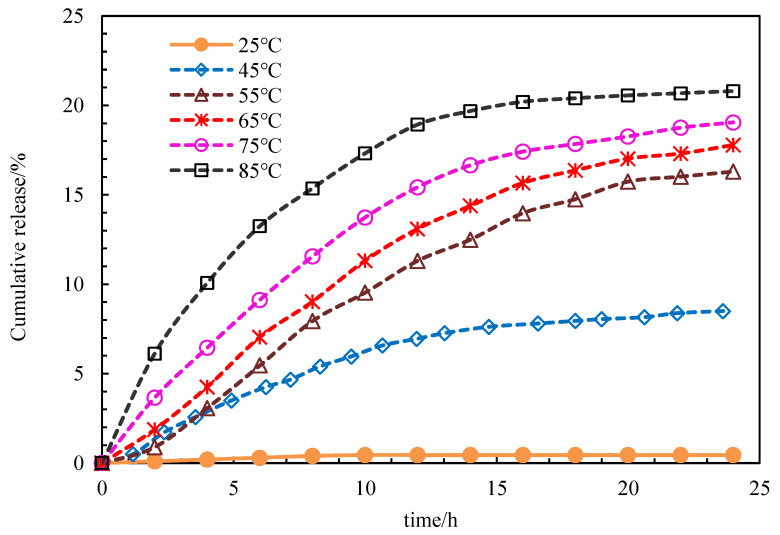
Changes in the cumulative release rate of the PG sample with time.

The experimental parameters are shown in Table 7. The release rate of PG samples with time at different temperatures was determined. The experimental conditions were a pH of 7 and a thickness of the PG shell of 14 nm. As shown in Figure 12, PG basically does not release at room temperature. With the increase in temperature, the release rate was accelerated and the release time was shortened.

### 3.3. Controlled Release Fitting

**Figure 13:**
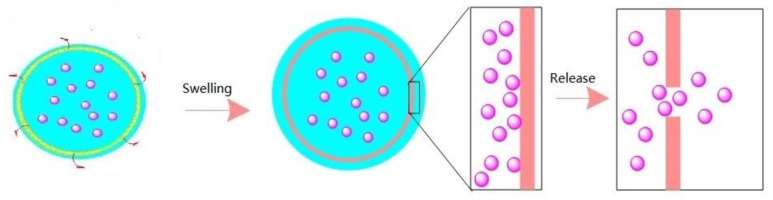
Flow chart of release.

A flow chart of the release is shown in Figure 13. Based on the fact that the prepared oil displacement nanocapsule may exhibit a variety of processes, the Ritger–Peppas equation was selected as the basic mathematical model:

(2) $$\frac{M_{t}}{M_{\infty }}=kt^{n}\;\frac{dM_{t}}{dt}=\frac{k}{t^{n}}$$

where $\frac{M_{t}}{M_{\infty }}$ is the cumulative release percentage at a certain time and *n* is the characteristic parameter of the release used to characterize the release mechanism. When *n* = 0.5 to 1, the release mode is mainly Fick diffusion and release, and the release speed is faster than Fick diffusion; when *n* = 1, the release is dominated by Fick diffusion, showing zero order dynamics. The release curve was fitted with Origin 8.0 software to observe the trend in variables.

Parameter settings:

Release medium: distilled water;

Speed: 50~150 rpm.

The experimental scheme was as follows: three datas taking points in the release rate were selected, the release time when the cumulative release rate reaches 90% was calculated, and it was fitted with a mathematical model according to the data of earlier data point. PG shows a slow-release state for shell degradation, and the initial membrane rupture and PG release law of slow-release is shown in the following (Equation (3):

(3) $$\frac{c(t)}{c_{\max}}=\frac{c_{i}}{c_{\max}}(1-e^{-\frac{t}{\tau_{1}}})+(1-\frac{c_{i}}{c_{\max}})(1-e^{-\frac{t}{\tau_{s}}})$$

$c_{\max}$ corresponds to the maximum concentration at 100% release;

$c_{i}$ corresponds to the initial membrane rupture concentration in a day;

$\tau_{1}$ is the characteristic time scale corresponding to initial membrane rupture;

$\tau_{s}$ is the characteristic time scale corresponding to continuous release.

**Figure 14:**
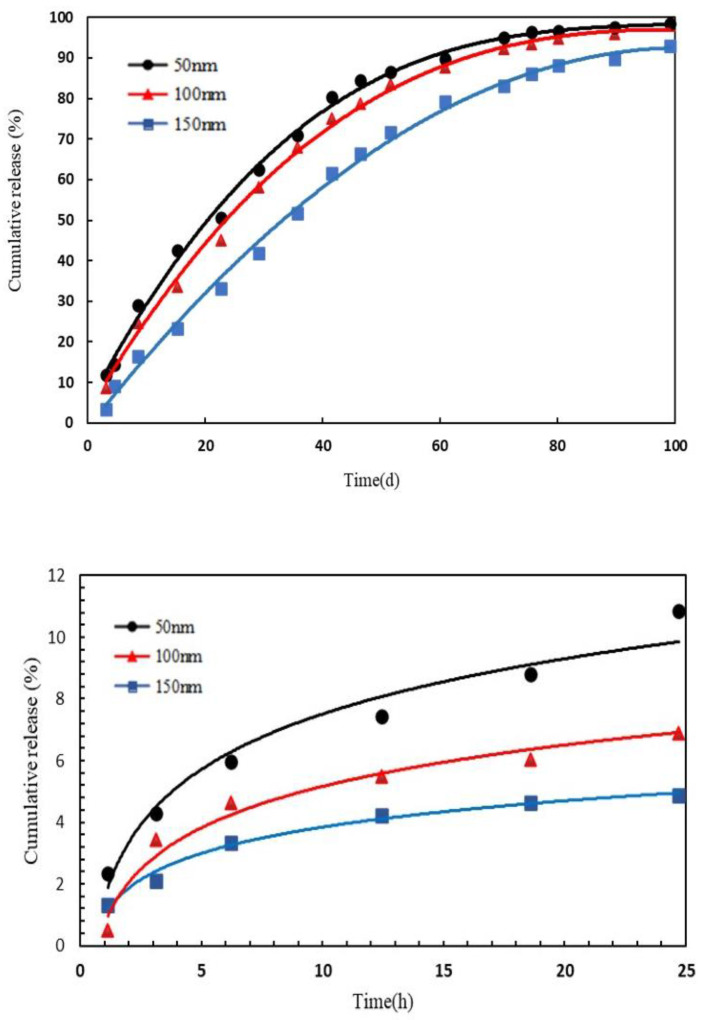
Curve of release rate of different sizes with time.

Corresponding to PG with different thicknesses, the concentration threshold is set differently, combined with the slow-release experimental data. The thickness was set as 50 nm, 100 nm and 150 nm and the $\frac{c_{i}}{c_{\max}}$ ratio of $\frac{c_{i}}{c_{\max}}$ corresponded to 0.15, 0.10 and 0.05. The fitting value,$\tau_{1}$, corresponds to 9 h, 10 h and 12 h. $\tau_{s}$ corresponds to 45d, 60d and 80d. If the release is highly continuous, the cumulative release time of 50% was 28 days, 43 days and 69 days. At 100 nm, the $\frac{c_{i}}{c_{\max}}$ ratio corresponds to 0.10, the pH was 7 and the cumulative release time of 50% was 43 days. The experimental data of the release rate of different PG particle sizes with time is shown in Figure 13. According to the formula calculation, the error rate was between 1.3% and 5.6%, so the release of capsules of particle size in the range of 50 nm to 150 nm conforms to the curve in Figure 14.

### 3.4. Oil Saturation Analysis of Core

In order to study the oil displacement dynamic characteristics of the nanocapsule system more intuitively, the change in oil-free saturation of the core was monitored during the experiment. Generally, the mixture of oil and gas is non-conductive. Water and crude oil differ greatly in electrical properties. The resistivity of crude oil is close to infinity, while the water in rocks is rich in metal salt ions. The greater the concentration of electrolyte ions, the smaller the resistance. Therefore, the change in oil saturation at the monitoring point can be determined according to the electrical change.

Use the theoretical method of Archie, formula [17], to calibrate the saturation, the saturation index *n* and lithology coefficient *b* were determined from the Archie formula, and the following formula was obtained for calculating water saturation:

(4) $$I=\frac{R_{t}}{R_{0}}=\frac{b}{S_{w}^{n}}$$

In the formula:

$R_{t}$ is the resistivity of rock with oil, Ω m;

$R_{0}$ is the resistivity of rock with complete water content, Ω m;

$S_{w}$ is the water saturation, %;

*b* is the lithological coefficient;

*n* is the saturation index.

**Figure 15:**
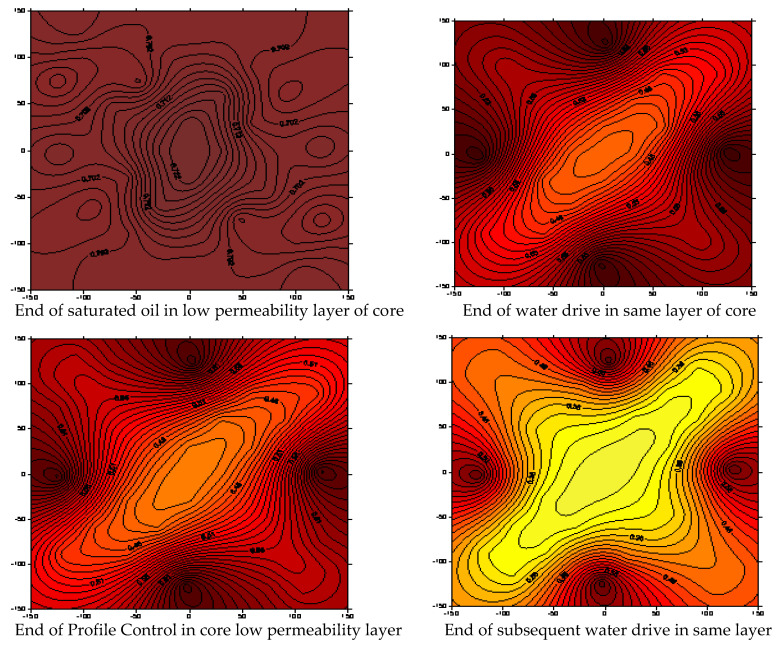
Core saturation field diagram.

There are two methods to determine the coefficients *b* and *n*: the core displacement experimental method and the empirical coefficient method. For pure sandstone, *b* = 1 and *n* = 2 are generally used. After several measurements, it was found that when the salinity reached 4000 or higher, the influence of the change in concentration change of the nanocapsule on the resistivity can be ignored. At this time, the resistivity of the monitoring point is mainly determined by the proportion of oil and water content at the monitoring location. The salinity of water used in this research experiment was 4500 mg/L, so the influence of the concentration on the water resistivity can be ignored. Software was applied to draw the oil saturation field variation diagram, and the experimental results are shown in Figure 15.

In heterogeneous cores, the residual oil in cores of low permeability layers is usually unaffected; therefore, the reservoir oil cannot be displaced. By analyzing the oil saturation field, it can be found that the nanocapsule displacement and the subsequent water flooding causes liquid absorption in the low permeability layer, expands the scope of coverage and decreases the residual oil saturation.

## 4. Conclusions

The surface chemical stability of a nanocapsule was analyzed by a potentiometric method. At 45 °C, the nanocapsule has changes in potential, which indicates that the nanocapsule is a slow-release nanocapsule at this temperature and the nanocapsule is temperature responsive. In the same release time, an alkaline or acidic environment can improve the release rate. When the temperature is in the range of 40~45 °C, the absorbance increases greatly. As the temperature continues to rise, the absorbance increases. Heating intensifies the surface shrinkage of the shell, increases the release amount and increases the release rate per unit time. In addition, the release time increases proportionally with the increase in shell thickness. The fitting data shows that the concentration threshold is set differently for nanocapsules with different thicknesses. The fitting values ($\tau_{1}$) correspond to 9 h, 10 h and 12 h. The fitting values ($\tau_{s}$) correspond to 45 days, 60 days and 80 days. If the release is highly continuous, the cumulative release time of 50% is 28 days, 43 days and 69 days. If the shell thickness is 100 nm, the cumulative release time of 50% under the condition of a pH of 7 is 43 days. Laboratory oil displacement experiments shows that the nanocapsule profile control and displacement agent can effectively spread the residual oil in the heterogeneous core and greatly reduce the oil saturation.

Outlook: Amphiphilic capsule-type profile control and displacement agents are a new type of amphiphilic nano-oil displacement agent with controllable size and temperature under specific reservoir temperature conditions. In this study, it is found that the capsule system can be used in low permeability reservoirs, as well as in high water content reservoirs with medium and high permeability. In the future, when the size reaches below 10 nm, it can be considered to be applied to reservoirs with lower permeability.

## Figures and Tables

**Table 1:** List of experimental parameters.

Concentration of PG (wt%)	Thickness of PG (nm)	Test Temperature (°C)	Salinity (mg/L)	pH Value	Hydration Time (h)
0.15	14	45	4000~20,000	7	36

**Table 2:** List of experimental parameters.

Concentration of PG (wt%)	Thickness of PG (nm)	Test Temperature (°C)	Salinity (mg/L)	pH Value	Hydration Time (h)
0.15	14	25, 45, 55, 65, 85	5000	7	36

**Table 3:** List of experimental parameters.

Concentration of PG (wt%)	Thickness of PG (nm)	Test Temperature (°C)	Salinity (mg/L)	pH Value	Hydration Time (h)
0.15	14	45	5000	5, 6, 7, 8	36

**Table 4:** List of experimental parameters.

Concentration of PG (wt%)	Thickness of PG (nm)	Test temperature (°C)	Salinity (mg/L)	pH Value	Hydration Time (h)
0.15	14, 32, 56, 96, 145	45	5000	7	24

**Table 5:** List of experimental parameters.

Concentration of PG (wt%)	Thickness of PG (nm)	Test temperature (°C)	Salinity (mg/L)	pH Value	Hydration Time (h)
0.15	56	45	5000	5, 7, 9	24

**Table 6:** List of experimental parameters.

Concentration of PG (wt%)	Thickness of PG (nm)	Test Temperature (°C)	Salinity (mg/L)	pH Value	Hydration Time (min)
0.15	14	40, 45, 55	5000	7	180

**Table 7:** List of experimental parameters.

Concentration of PG (wt%)	Thickness of PG (nm)	Test Temperature (°C)	Salinity (mg/L)	pH Value	Hydration Time (h)
0.15	14	25, 45, 55, 65, 75, 85	5000	7	24

## Data Availability

The data presented in this study are available in the article.
